# Development and Validation of Quantitative Real-Time PCR for the Detection of Residual CHO Host Cell DNA and Optimization of Sample Pretreatment Method in Biopharmaceutical Products

**DOI:** 10.1186/s12575-019-0105-1

**Published:** 2019-09-01

**Authors:** Weifeng Zheng, Lin Jiang, Qing Lei, Jun Yang, Xuefeng Gao, Wanru Wang, Yanli Zhang, Tao Kong, Qiaoli Chen, Gang Li

**Affiliations:** The fourth research department, Lanzhou Institute of Biological Products Co., Ltd, No.888 Yanchang Road, Chengguan District, Lanzhou City, Gansu Province China

**Keywords:** Residual DNA, qPCR, CHO

## Abstract

**Background:**

The presence of residual DNA carried by biological products in the body may lead to an increased oncogenicity, infectivity, and immunomodulatory risk. Therefore, current agencies including WHO, EU, and the FDA limited the accepted amounts of residual DNA (less than 10 ng or 100 pg/dose). Among the methods of detecting residual DNA, qPCR is considered to be the most practical for residual DNA quantitation due to its sensitivity, accuracy, precision, and time-saving.

**Results:**

In this study, the detection capacity of this method was determined by comparing the detected concentration of the commercial kit and the self-designed primer/probe set after the same treatment of the extraction method. Then, a universal sample pretreatment method based on a co-precipitant was optimized. The validation results demonstrated that the method has appropriate specificity, sensitivity, accuracy, and precision according to ICH guidelines. The limit of detection and quantitation reached 3 fg/ul and 0.3 pg/reaction respectively, which satisfies the requirement of limit of residual DNA detection in biologics. Spike recovery (82.3–105.7%) showed that the proposed qPCR assay was accurate and has good extraction efficiency. Moreover, the precision of the method based on intra- and inter-assay was 0.065–0.452% and 0.471–1.312%, respectively.

**Conclusions:**

These results all indicated that the method for determination of residual DNA in biological products expressed from CHO cells is sensitive, accurate and robust.

**Electronic supplementary material:**

The online version of this article (10.1186/s12575-019-0105-1) contains supplementary material, which is available to authorized users.

## Background

Biological products such as recombinant protein, antibody and vaccine are all expressed from the hosts of bacterial, yeast, animal cells, and continuous cell lines in the process of production, such as recombinant hepatitis B vaccine(CHO cell), Vero cell rabies vaccine, monoclonal antibody and some recombinant therapeutic proteins [1–5]. The products still contains fragments of DNA from the host cells even after conducting a rigorous purification process. The presence of these residual DNA molecules in the body along with biological products may lead to increased oncogenicity, infectivity, and immunomodulatory risk [6–8], and it is estimated that the probability of the residual DNA integrating into the genome and inducing cancer is 10^–10^ [9, 10]. Hence, the World Health Organization(WHO) and European Union(EU) allow the amounts for up to 10 ng/dose of residual DNA, and the US Food and Drug Administration(FDA) allow it for up to 100 pg/dose [11–13]. Highly sensitive and accurate methods for detection and quantitation of low level DNA are needed to meet this requirement.

Three methods(DNA hybridization assay, Threshold® assay, and quantitative PCR)have been recommended by regulatory agencies for residual host cell DNA quantitation [14, 15]. Among these methods, qPCR is considered to be the most practical for residual DNA quantitation due to their sensitivity, accuracy, precision, and time-saving features. It has successfully developed some kits for quantitatively detecting the residual DNA of *Escherichia coli*, NS0, yeast, and Chinese hamster ovary(CHO) cells [16]. The current qPCR kits are all based on solid-phase, liquid-phase or magnetic beads to extract the residual host DNA from samples for quantification. Although there are some methods that can directly detect residual DNA by no-extraction, these methods may result in great differences due to its applicability for different proteins [17, 18].

The objective of this paper is to develop a method for detecting the residual CHO cell DNA based on TaqMan Real-Time PCR. The method is cost-effective and more conveniently used to guide the downstream purification process, as well as to improve the production process and the standard of safety quality control.

## Results

### Comparison of Detected Concentration between the Kit and the Alu-Primers/Probe

The overall experimental conditions of qPCR were optimized (data not shown) after the primer/probe was designed against the Alu sequence. After the samples were treated with PrepSEQ Residual DNA Sample Preparation Kit, qPCR was conducted using the resDNASEQ Quantitative CHO DNA Kit and Alu-primer/probe. The differences of concentrations detected were compared after standardization according to the recovery rate. The standardized residual DNA concentration of the Kit and Alu-primer/probe were 0.485 pg/ml and 0.577 pg/ml, respectively(Table 1). The same comparison was made on four different samples. The results were shown in Table 2.

**Table 1:** Comparison of detected concentration after standardization

	observed concentration(pg/ml)			Recovery(%)	Standardized DNA concentration(pg/ml)
Kit	0.337	0.51	0.365	83.3	0.485
Alu-primer/probe	0.331	0.478	0.485	74.7	0.577

**Table 2:** Comparison of the detected concentration of four samples

Sample	Kit(pg/ml)	Alu-primer/probe(pg/ml)
A	UD^a^	0.534
B	UD	0.893
C	UD	UD
D	0.163	0.283

^a^*UD* undetectable

### Sample Pretreatment Method Optimization

**Fig. 1:**
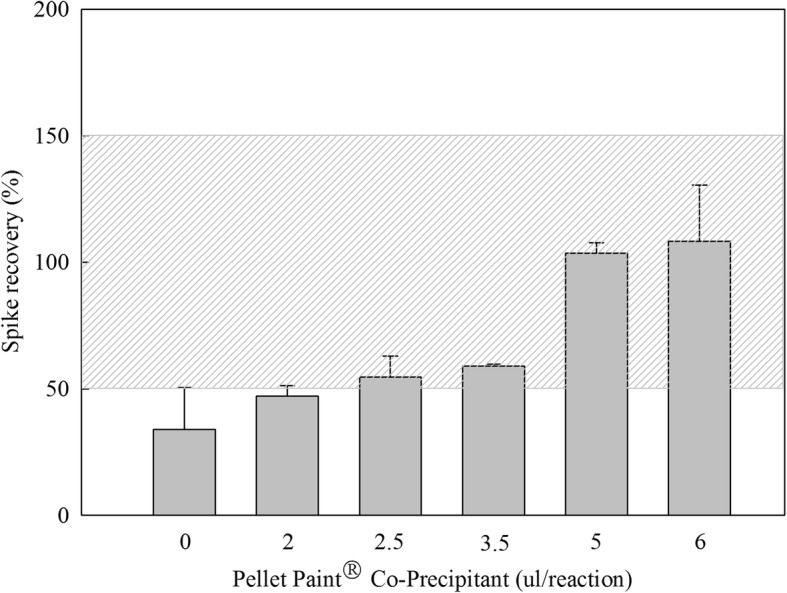
The change of DNA spike recovery. The error bar represents the standard deviation, the shadow zone represents the acceptance criteria (50–150%) of spike recovery

Protein samples were digested by protease K(2 mg/ml) for SDS-PAGE at different temperatures and treatment time. Subsequently the processing temperature and time were determined by observing the size and number of bands(the smaller or less the bands, the better the digestion effect of protease K), while excessive protease K was removed by subsequent steps(data not shown). The host cell residual DNA was precipitated by Pellet Paint® Co-Precipitant prior to detection to avoid the interference of proteins or other components in the sample. Observing the recovery rate change by continually changing the amount of Pellet Paint® Co-Precipitant. As the amount of Pellet Paint® Co-Precipitant increased, the recovery rate gradually stabilized to about 100%(Fig. 1). The centrifugal speed in addition to other steps were also optimized.

### Specificity

**Fig. 2:**
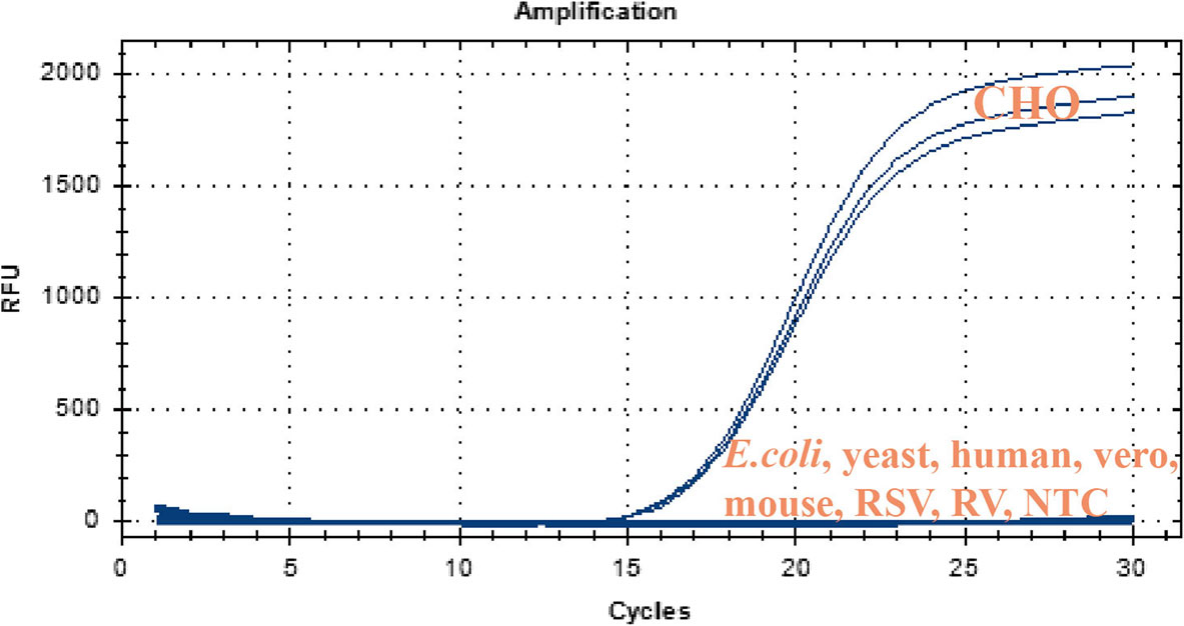
Specificity test. Only the CHO genome has an amplification curve

The amplicon did not overlap with the genomes of other species by BLAST analysis. Then the 10 pg/ul genomic DNA of CHO, E.coli, yeast, human, vero cell, mouse, respiratory syncytial viral(RSV), and rabies virus(RV) were amplified with Alu-primer/probe. The primer and probe did not amplify irrelevant genome DNA sequences. It can be seen from the figure that only the CHO genome was amplified while the others and the no-template control(NTC) were not. (Fig. 2).

### LOD and LOQ

**Fig. 3:**
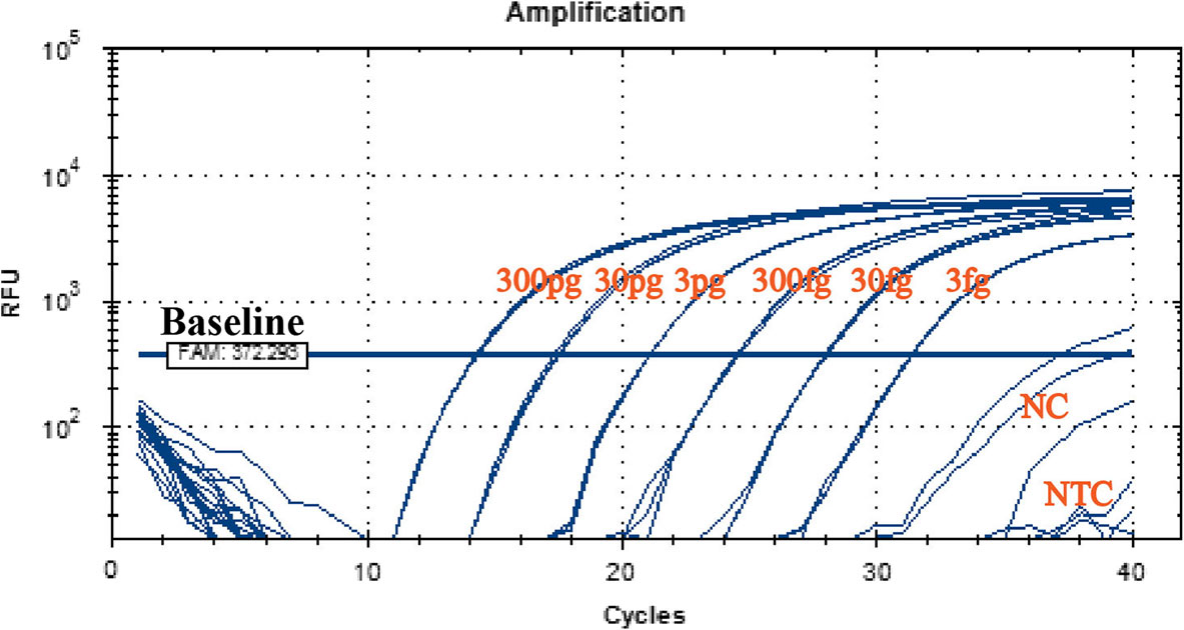
The determination of detection limit. All standards(3 fg/ul ~ 3 × 10^6^ fg/ul) had an amplification curve with good repeatability. NC represents the negative control, NTC represents the no-template control. Horizontal lines in the figure refer to the baseline, which is determined by the software itself

The LOD (limit of detection), the analyte can be detected reliably, was determined by establishing the standard curve. The ranges of the standard curve of CHO genomic DNA were 3 fg/ul ~ 3 × 10^6^ fg/ul, each standard was tested in triplicate, all of which were detectable by the assay (Fig. 3). 3 and 0.3 pg of CHO DNA standard were added to the protein samples (150ul), and the qPCR was performed after extraction using the co-precipitation method. We observed whether it could be accurately measured with an appropriate recovery rate to determine LOQ (limit of quantitation). The experimental results showed that the LOD and LOQ of the assay were at least 3 fg/ul and 0.3 pg/reaction for CHO DNA, respectively (Table 3).

**Table 3:** Limit of quantification(LOQ)test

Spike amount (pg/reaction)	Mean C_T_^*^	Mean value of observed DNA (pg/reaction)	Standard deviation	Average Recovery(%)
3	31.98	3.14	0.298	105
0.3	35.31	0.294	0.173	98

^*^C_T_, Cycle Threshold

### Precision

Intra-assay precision was determined from experiment results in an individual run. The standard deviation(SD) and coefficient of variation(CV) of each standard were 0.014–0.145 cycles and 0.065–0.452%(Table 4).Inter-assay precision was determined from experiment results in three different runs on three different days. The SD and CV were 0.118–0.190 cycles and 0.471–1.312%, respectively(Table 5).

**Table 4:** Intra-assay precision(repeatability)test

Standard(pg/ml)	Mean C_T_(*n* = 3)	Standard Deviation	C.V.(%)
300	14.50	0.021	0.145
30	17.78	0.015	0.084
3	21.41	0.014	0.065
0.3	25.05	0.062	0.248
0.03	28.5	0.061	0.214
0.003	32.09	0.145	0.452

**Table 5:** Inter-assay precision(intermediate precision)test

Standard(pg/ml)	Mean C_T_			Standard deviation	C.V.(%)
	Day1	Day2	Day3		
300	14.50	14.3	14.73	0.190	1.312
30	17.78	17.53	17.9	0.179	1.011
3	21.41	21.12	21.49	0.165	0.772
0.3	25.05	24.83	25.00	0.118	0.471
0.03	28.5	28.21	28.42	0.141	0.497
0.003	32.09	31.76	31.82	0.188	0.591

### Accuracy

Three different concentrations(600 pg/ml, 200 pg/ml, 20 pg/ml) were obtained by adding different amounts of CHO DNA into the protein samples. The spiked samples were processed by the optimized sample pretreatment method based on co-precipitant and then the qPCR experiment was carried out by 9 replicates on different days. Then the extraction effect was evaluated by calculating the recovery rate. The average percentage recovery was 82.3–105.7% (Table 6) with a CV of < 25%. These results also showed that the optimized sample pretreatment method had a good extraction efficiency.

**Table 6:** Accuracy test by observing recovery of different spike concentrations

Spiking concentration(pg/ml)	Measured(pg/ml)	Average recovery(%)	CV(%)
600	494 ± 19.8	82.3	4.0
200	206.7 ± 7.9	103.3	3.8
20	21.1 ± 4.5	105.7	21

### Linearity

**Fig. 4:**
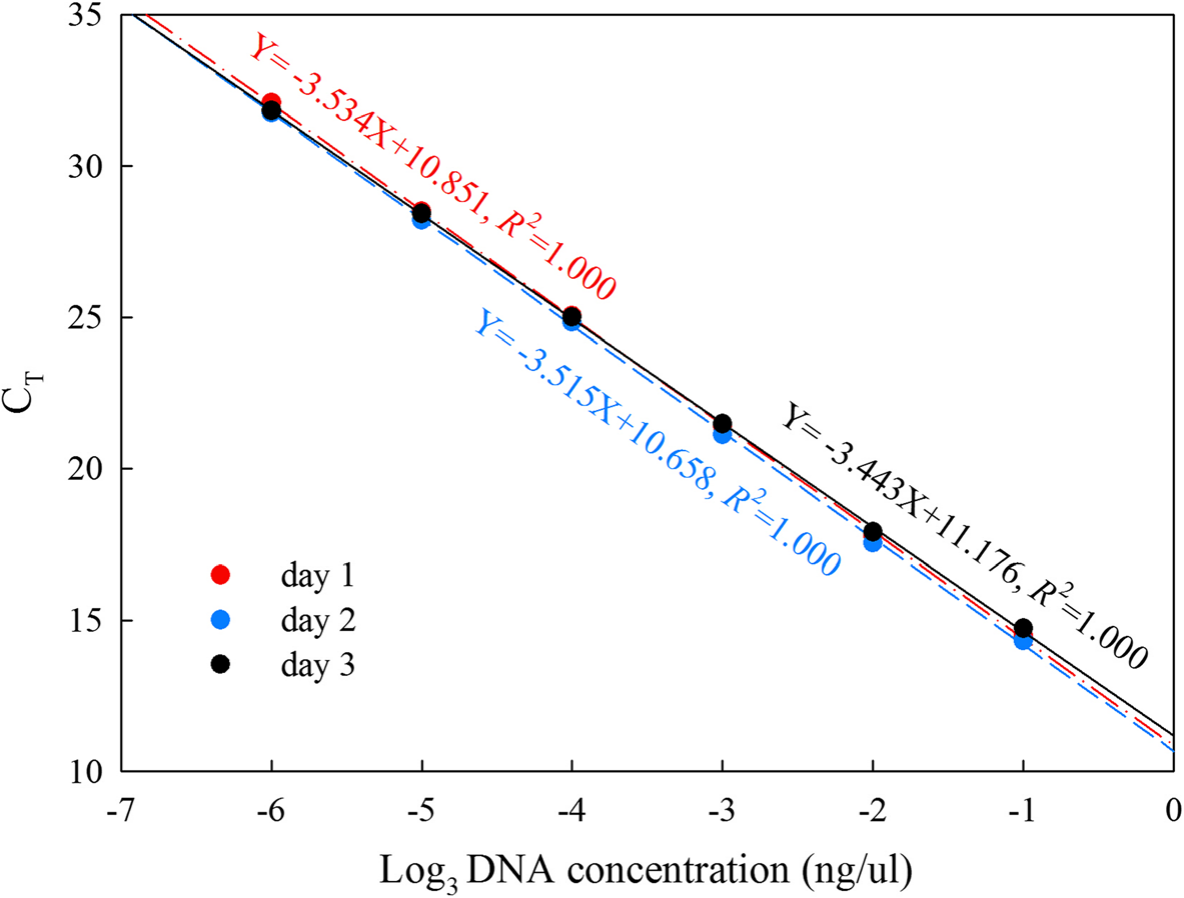
Standard curves of three independent assays. Red, blue, and black represent the standard curves of day1, day2, and day3, respectively

The linearity of this method was evaluated by calculating coefficients of determination(R^2^). Three standard curves were created by plotting mean C_T_ versus the DNA concentration. Standard curves were obtained from three independent assays performed in different days. The R^2^ of the standard curves reached 1.000 (Fig. 4),which meets the accepted criterion of R^2^ > 0.98.

## Discussion

Recombinant proteins have been used to treat different diseases in recent years, among which monoclonal antibodies are the most rapidly developed. CHO cells are the most widely used cell lines for the production of these recombinant proteins. A reliable and sensitive qPCR method for detecting the residual DNA of host cells is required for biological products expressed from CHO cell. The use of commercial kits, to some extent, increased the cost of detection, and the complex operation process can easily impact the stability of experimental results. Therefore, in this study, the detection capacity was determined by comparing the detection amount of commercial kits to the designed primer/probe. Then the sample pretreatment method based on the co-precipitant was established and optimized, and the extraction effect was also tested, so as to establish a qPCR method that is not inferior to commercial kits.

Highly repetitive Alu-equivalent sequences in CHO genome was selected to design primers and probes, thus to maximize matches to the templates and to improve sensitivity. The detected concentration comparison results in Table 1 and Table 2 show that Alu-primer/probe has good detection capacity.

Detection of residual DNA requires accurate quantification of picogram levels of DNA in mg (or larger) quantities of product, which may exists in a variety of matrices. When the product or other sample components interfere with the assay results, dilution may be all that is required to overcome the interference, so long as the specified DNA content of the sample remains within the useful range of the analytical procedure. But when sample dilution is not effective in reducing assay interference, it is necessary to use a sample pretreatment procedures. Existing residual DNA detection methods, based on qPCR, typically rely on the extraction of the residual DNA from protein samples prior to residual DNA quantification to avoid interference of protein and other components. Although the most of extraction approaches use solid phase DNA extraction procedures based on magnetic particle or liquid phase based on sodium iodide, these approaches require too much reagents and the steps are cumbersome, making the experiment extremely time-consuming.

Here, we described a universal sample pretreatment method based on co-precipitant (theoretically applicable to all protein samples): At the appropriate pH, the protein is digested by proteinase K to release residual DNA molecules. The residual DNA is then combined with co-precipitant and separated by centrifugation. Excess protein K is also dissolved in isopropanol and removed by centrifugation. DNA precipitation was washed with 70% ethanol to remove residual isopropanol, protein and salt ions. Finally, it was dissolved in TE buffer for residual DNA quantification. The process-intermediate samples with different buffers were processed and tested by the method, and the results are shown in Additional file 1: Table S1 (69.2–120%). The results in Table 6 also show that this method has a good extraction efficiency. It should be considered that this extraction protocol may not be suitable for all samples, and the differences in sample properties may result in a difference in recovery. When sample characteristics (e.g., matrix effects or sample pretreatment method) make achieving a recovery acceptance criterion of 50%~ 150% impractical, then correcting the observed DNA concentration by using the load recovery percentage is also an acceptable approach [19].

## Conclusion

The optimized assay was further validated according to the ICH guideline [19] for testing CHO cell derived residual DNA. The sensitivity, accuracy, and precision of the qPCR assay were optimized using the Chinese hamster Alu-equivalent type 2 repeat gene. The LOD of TaqMan qPCR assay reached 3 fg/ul, this showed that the assay has good sensitivity. In the spike recovery study, the analytical results (82.3–105.7%) showed that the proposed qPCR assay was accurate. The LOQ reached approximately 0.3 pg/reaction, which satisfies the requirement of limit of residual DNA detection in biologics. The validation results were summarized in Table 7.

**Table 7:** Summary of the validation of qPCR for CHO residual DNA

Results		
Valid method range		3 fg/ul-300 pg/ul
Linearity		1.000
LOD		3 fg/ul
LOQ		0.3 pg/reaction
Accuracy		82.3–105.7%
Precision	Repeatability(intra-precision)	0.065–0.452%
	Inter- precision	0.471–1.312%

In this study, a sensitive, reliable, and precise method for residual DNA quantitation in the CHO expression system was developed and optimized, which is not inferior to commercial kits in detection capability by comparison. Based on the development strategy of this study, it is easier to develop qPCR method for residual host DNA for other expression systems such as E.coli, yeast, and vero cell.

## Methods

### Standards Preparation

CHO Genomic DNA was extracted from fresh CHO-k1 cell cultures using the QIAamp® DNA Mini kit(Qiagen, Germany)according to manufacturer’s protocol including RNAse lysis step. Eluted DNA was quantified by UV spectrophotometry at an absorbance of 260 nm and 280 nm and it was stored at − 20 °C for later use.

### DNA Extraction

The PrepSEQTM Residual DNA Sample Preparation Kit (Applied Biosystems, USA) based on magnetic particle was used to extract residual DNA from therapeutic proteins expressed by CHO cells according to manufacture’s protocol. However, this method based on magnetic particle can easily cause DNA loss due to the complicated operation steps, which produces different experimental results .

In order to simplify the process of experimental operation and improve the stability of the experiment. We developed a new sample pretreatment method based on co-precipitant(Merck, Germany): 150 ul of protein samples were diluted three times with TE buffer(Sangon Biotech, China) to maintain the pH in the activity ranges(pH 6–8) of Proteinase K(Sangon Biotech, China), then were spiked with and without 10 ul of CHO DNA standard. Samples, spiked samples and Negative control (450ul TE buffer) were incubated at 60 °C with 25ul of proteinase K(2 mg/ml) for 60 min. Residual DNA was precipitated by 5 ul Pellet Paint® Co-Precipitant(Merck, Germany), which is a visible/fluorescent dye-labeled carrier formulated specifically for use in alcohol precipitation of nucleic acid, 50 ul 5 M NaCl and 500ul isopropanol(Sinopharm Chemical Reagent, China) at room temperature for 5 min, then recovered by centrifugation at 12000 rpm for 10 min. The DNA precipitate was washed with 70% ethanol(Sinopharm Chemical Reagent, China) and centrifuged at 12000 rpm for 8 min, dried at room temperature for 30 min and dissolved in TE buffer for qPCR.

### Primers and Probe

Primer/probe sets were designed against the Chinese hamster Alu-equivalent sequences using online tool PrimerQuest (https://sg.idtdna.com/pages)(Integrated DNA Technologies, IDT, Coralville,IA,USA).Primer/probe were evaluated by software Oligo7 including duplex formation, hairpin formation and Amplicons secondary structure, etc. All primer and probe were custom-synthesized and HPLC-purified at Sangon Biotech(China).The primer/probe set was:forward primer:5′-AGAGATGGCTCGAGGTTAAG-3′, reverse primer: 5′-TCTGCACACCAGAAGAGG-3′, probe: 5′-6-FAM-AGCACCAACTGCTCTTCCAGAGG-BHQ1–3′.

The total volume of 20 ul reaction system included the following: 10 ul of TaqProbe 2X qPCR-Multiplex(Sangon Biotech, China), 0.4 ul of each of the forward and reverse primer, 0.2 ul of TaqMan probe, and 8.6 ul of standard, samples and spiked samples that were extracted, negative control and No template control. All samples were analyzed in triplicate replicate wells. The qPCR was performed with CFX96TM Real-Time System(BIO-RAD, USA)using the following thermal cycling conditions:initial heat denaturation at 95 °C for 10 min, followed by 40 cycles each consists of 95 °C for 15 s and 58 °C for 1 min.

### Detected Concentration Contrast

In order to compare the detected concentration of Alu-primers/probe and the resDNASEQ™ Quantitative CHO DNA Kit(Applied Biosystems, USA) on the same sample, the extracted DNA from samples by The PrepSEQTM Residual DNA Sample Preparation Kit was tested by qPCR. This were done to determine the detection capacity of Alu-primer/probe. The commercial extraction kits were only used to determine the detection capacity of Alu-primers/probe, subsequent methodological validation or other experiments used the co-precipitation method that was mentioned above.

### Validation Study and Acceptance Criteria

The specificity, LOD, LOQ, precision, accuracy and linearity of the assay for quantitative detection of residual CHO DNA were validated according to the International Conference on Harmonisation (ICH) guideline [20]. To determine the specificity, the BLAST analysis was performed in the NCBI to observe whether the amplicon sequence and other species duplicated. Subsequently, CHO and other genomic DNA (10 ng/ml) were used to perform qPCR to observe the amplification curve. The LOD was determined by establishing a standard curve and the linearity was also verified. A standard curve was generated by plotting the logarithm of the concentration of standard DNA against the threshold cycle(CT). LOQ is lowest amount of analyte in the sample, which can be quantitatively determined with suitable precision and accuracy. Furthermore, it was determined by suitable spike recovery. Precision study was used to assess repeatability(intra-assay precision) and intermediate precision. Inter-assay and intra-assay variability were quantified by three independent analyses on different days. The accuracy of the method was determined by the spike recovery calculated by adding different concentration of genomic DNA. Our assay acceptance criteria was as follows: the accuracy of 50–150%, intra-assay precision of ≤5%, inter-assay precision of ≤10%, a linearity of the standard curve of R^2^ ≥ 0.98.

## Additional file

Additional file 1: **Table S1.** DNA spike recovery in process-intermediate samples. (DOCX 19 kb)

## Data Availability

The data that support the findings of this study are available from the corresponding author upon reasonable request.

## Abbreviations

CHO: Chinese hamster ovary cells
CT: Cycle Threshold
CV: Coefficient of variation
EU: European Union
FDA: Food and Drug Administration
LOD: Limit of detection
LOQ: Limit of quantitation
NTC: No template control
RSV: Respiratory syncytial viral
RV: Rabies virus
SD: Standard deviation
UD: Undetectable
WHO: World Health Organization

## References

[1] Hess RD, Weber F, Watson K, Schmitt S (2012). Regulatory, biosafety and safety challenges for novel cells as substrates for human vaccines. Vaccine..

[2] Koudstaal W, Hartgroves L, Havenga M, Legastelois I, Ophorst C, Sieuwerts M, Zuijdgeest D, Vogels R, Custers J, de Boer-Luijtze E, de Leeuw O, Cornelissen L, Goudsmit J, Barclay W (2009). Suitability of PER.C6 cells to generate epidemic and pandemic influenza vaccine strains by reverse genetics. Vaccine..

[3] Wu X, Smith TG, Rupprecht CE (2011). From brain passage to cell adaptation: the road of human rabies vaccine development. Expert Rev Vaccines.

[4] Petricciani J, Sheets R (2008). An overview of animal cell substrates for biological products. Biologicals..

[5] Sanders BP, Edo-Matas D, Custers JH, Koldijk MH, Klaren V, Turk M, Luitjens A, Bakker WA, Uytdehaag F, Goudsmit J, Lewis JA, Schuitemaker H, PER.C6(®) cells as a serum-free suspension cell platform for the production of high titer poliovirus: a potential low cost of goods option for world supply of inactivated poliovirus vaccine, Vaccine. 31(2013):850–856. 10.1016/j.vaccine.2012.10.070. 10.1016/j.vaccine.2012.10.070 23123018

[6] Sheng-Fowler L, Tu W, Fu H, Murata H, Lanning L, Foseh G, Macauley J, Blair D, Hughes SH, Coffin JM, Lewis AM, Peden K (2014). A mouse strain defective in both T cells and NK cells has enhanced sensitivity to tumor induction by plasmid DNA expressing both activated H-Ras and c-Myc. PLoS One.

[7] Sheng-Fowler L, Lewis AM, Peden K (2009). Quantitative determination of the infectivity of the proviral DNA of a retrovirus in vitro: evaluation of methods for DNA inactivation. Biologicals..

[8] André M, Reghin S, Boussard E, Lempereur L, Maisonneuve S (2016). Universal real-time PCR assay for quantitation and size evaluation of residual cell DNA in human viral vaccines. Biologicals..

[9] Sheng L, Cai F, Zhu Y, Pal A, Athanasiou M, Orrison B, Blair DG, Hughes SH, Coffin JM, Lewis AM, Peden K (2008). Oncogenicity of DNA in vivo: tumor induction with expression plasmids for activated H-ras and c-myc. Biologicals..

[10] Sheng-Fowler L, Lewis AM, Peden K (2009). Issues associated with residual cell-substrate DNA in viral vaccines. Biologicals..

[11] WHO. WHO expert committee on biological standardization: fifty-fourth report: World Health Organization; 2007. http://www.who.int/bloodproducts/catalogue/en/. Accessed 1 Oct 2018.

[12] Knezevic I, Stacey G, Petricciani J, Sheets R; WHO Study Group on Cell Substrates, Evaluation of cell substrates for the production of biologicals: Revision of WHO recommendations. Report of the WHO Study Group on Cell Substrates for the Production of Biologicals, 22-23 April 2009, Bethesda, USA, Biologicals. 38(2010):162–169. 10.1016/j.biologicals.2009.08.019. 10.1016/j.biologicals.2009.08.019 19818645

[13] FDA (1997). Point to consider in the manufacture and testing of monoclonal antibody antibody products for human use.

[14] Cai H, Gu X, Scanlan MS, Lively CR (2011). Development of a quantitative PCR assay for residual mouse DNA and comparison of four sample purification methods for DNA isolation. J Pharm Biomed Anal.

[15] Zamanian S, Mohammadi-Yeganeh S, Kia V, Kaghazian H, Paryan M (2017). Design and validation of a new method to detect and quantify residual host cell DNA in human recombinant erythropoietin (rEPO). Prep Biochem Biotechnol.

[16] Chen Z, Dai H, Liu Z, Zhang L, Pang J, Ou J, Yang D (2014). Quantitation of the residual DNA from rice-derived recombinant human serum albumin. Anal Biochem.

[17] Peper G, Fankhauser A, Merlin T, Roscic A, Hofmann M, Obrdlik P (2014). Direct real-time quantitative PCR for measurement of host-cell residual DNA in therapeutic proteins. J Pharm Biomed Anal.

[18] Hussain M (2015). A direct qPCR method for residual DNA quantification in monoclonal antibody drugs produced in CHO cells. J Pharm Biomed Anal.

[19] The United States Pharmacopeia Convention, General Information <1130>: Nucleic Acid-Based Techniques—Approaches For Detecting Trace Nucleic Acids (Residual Dna Testing), in: USP40-NF35, 2016, pp.1507–1511.

[20] Borman P, Elder D, Teasdale A, Elder D, Nims RW (2018). Q2(R1) validation of analytical procedures: text and methodology. ICH Quality Guidelines: An Implementation Guide.

