# Supplementary material for: Development and Validation of Quantitative Real-Time PCR for the Detection of Residual CHO Host Cell DNA and Optimization of Sample Pretreatment Method in Biopharmaceutical Products
Source: Biol Proced Online. 2019 Sep 1;21:17. doi: 10.1186/s12575-019-0105-1 (PMC6717637; doi:10.1186/s12575-019-0105-1)
Supplement: Supplementary file 1 — Table S1. DNA spike recovery in process-intermediate samples. (DOCX 19 kb) [file 12575_2019_105_MOESM1_ESM.docx]

Table 1 DNA spike recovery in process-intermediate samples.

| Sample | Result (pg/ml) | Average Result (pg/ml) | Spike (pg) | Average Spike | C_T_ | CV% | Spike Recovery | Reported Results (pg/mg) |
| --- | --- | --- | --- | --- | --- | --- | --- | --- |
| Protein A Pool（7.58 mg/ml, a^*^） | 5.61E+04 | 5.71E+04 | 2514 | 2511.7 | 20.2  ±  0.032 | 0.158 | 83.7% | 7.53E+03 |
|  | 5.68E+04 |  | 2212 |  |  |  |  |  |
|  | 5.83E+04 |  | 2809 |  |  |  |  |  |
| Cation Exchange (6.86 mg/ml, b^*^) | 2.23E+04 | 2.35E+04 | 2832 | 2808.7 | 21.6  ±  0.073 | 0.338 | 93.7% | 3.43E+03 |
|  | 2.37E+04 |  | 2690 |  |  |  |  |  |
|  | 2.48E+04 |  | 2904 |  |  |  |  |  |
| Cation Exchange-Cleaning (6.24 mg/ml, c^*^) | 2.02E+05 | 2.02E+05 | 252 | 340.3 | 18.22  ±  0.023 | 0.126 | 113.3% | 3.24E+04 |
|  | 1.99E+05 |  | 485 |  |  |  |  |  |
|  | 2.05E+05 |  | 284 |  |  |  |  |  |
| Anion Exchange-Elution (18.76 mg/ml, d^*^) | 2.32 | 3.05 | 19.5 | 20.7 | 35.71  ±  0.386 | 1.081 | 69.2% | 0.163 |
|  | 3.79 |  | 20.0 |  |  |  |  |  |
|  | 3.04 |  | 22.8 |  |  |  |  |  |
| Anion Exchange-Cleaning (6.39 mg/ml, c^*^) | 5.60E+04 | 5.82E+04 | 3102 | 3716.3 | 19.13  ±  0.05 | 0.261 | 120% | 9.108E+03 |
|  | 5.95E+04 |  | 3339 |  |  |  |  |  |
|  | 5.92E+04 |  | 4708 |  |  |  |  |  |

*a, b, c, d represents different buffers.
